# Bioactive Lactones from the Mangrove-Derived Fungus *Penicillium* sp. TGM112

**DOI:** 10.3390/md17080433

**Published:** 2019-07-24

**Authors:** Meng Bai, Guo-Lei Huang, Rong-Qing Mei, Bin Wang, You-Ping Luo, Xu-Hua Nong, Guang-Ying Chen, Cai-Juan Zheng

**Affiliations:** 1 Key Laboratory of Tropical Medicinal Resource Chemistry of Ministry of Education, College of Chemistry and Chemical Engineering, Hainan Normal University, Haikou, Hainan 571158, China; 2 Key Laboratory of Tropical Medicinal Plant Chemistry of Hainan Province, Hainan Normal University, Haikou, Hainan 571158, China

**Keywords:** *Penicillium* sp., lactones, antibacterial activity, insecticidal activity

## Abstract

Three new lactones penicilactones A−C (**1**−**3**) were obtained from the mangrove-derived fungus *Penicillium* sp. TGM112. Their structures and absolute configurations were determined by detailed NMR, MS spectroscopic data, Mo_2_(OAc)_4_-induced electronic circular dichroism (ECD), and circular dichroism (CD) spectroscopy. Compound **1** showed antibacterial activity against *Staphylococcus aureus* with an MIC value of 6.25 μg/mL. Compound **2** showed insecticidal activity against newly hatched larvae of *Culex quinquefasciatus* with the LC_50_ value of 78.5 (±0.58) μg/mL.

## 1. Introduction

**Figure 1:**
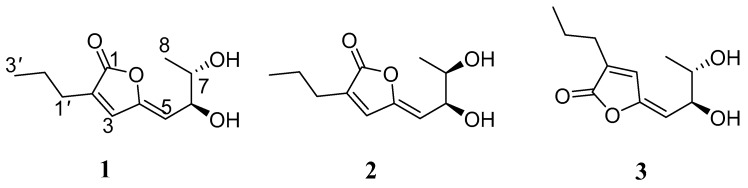
The structures of compounds **1**–**3.**

Natural products from marine-derived fungi have played an important role for leading compounds and have attracted interests from chemists and biologists [1]. An increasing number of structural novel and bioactive natural products have been obtained from mangrove-derived fungi [2]. Ordinarily, lactones with a 6,7-dihydroxyocta-2,4-dien skeleton were a small group of natural products. To date, only 12 ones, including cytotoxic goniobutenolodes A and B [3], 6,7-dihydroxy-2-propyl-2,4-octadien-4-olide [4], versicolactones A and B [5], antioxidant litchiol B [6], and plecmillins G-L [7], have been identified from natural resources. Moreover, goniobutenolodes A and B [8], versicolactones A and B and six stereoisomers [9], have been synthesized. As part of our continuing investigation into bioactive metabolites of mangrove-derived fungi [10,11,12,13], two new meroterpenoids and eight new isocoumarins have been identified from the fermentation of *Penicillium* sp. TGM112, a fungus obtained from the mangrove *Bruguiera sexangula var*. *rhynchopetala* [14]. Furthering our work on the remaining extract of the fungal strain *Penicillium* sp. TGM112, it was found that its fermentation extract showed antibacterial activity against *Staphylococcus aureus* with an MIC value of 20 μg/mL. The subsequent bioassay-guided fractionation and isolation were carried out and resulted in the characterization of three new lactone penicilactones A−C (**1**−**3**) (Figure 1). Herein, the isolation, structure elucidation, and bioactivities of these compounds are described.

## 2. Results and Discussion

### 2.1. Structure Elucidation

**Figure 2:**
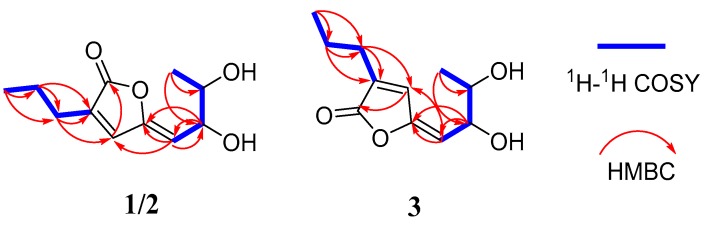
^1^H-^1^H COSY correlations and key HMBC correlations for compounds **1**–**3.**

**Figure 3:**
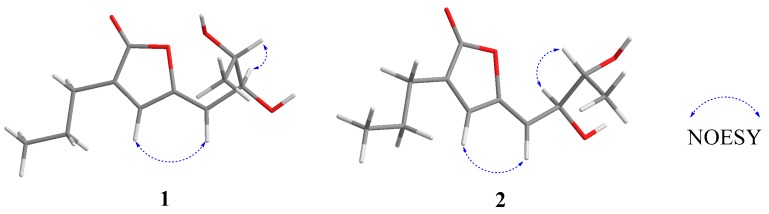
Key NOESY correlations for compounds **1** and **2**.

Compound **1** was isolated as a yellow oil, with the molecular formula of C_11_H_16_O_4_ (four degrees of unsaturation) determined by the HRESIMS data. The ^1^H NMR data (Table 1 and Figure S1) showed two methyl signals at *δ*_H_ 1.13 (3H, d, *J* = 6.8 Hz, H-8) and 0.98 (3H, t, *J* = 7.2 Hz, H-3′), two methylene signals at *δ*_H_ 2.31 (2H, t, *J* = 6.8 Hz, H-1′) and 1.60 (2H, m, H-2′), two oxygenated methine groups at *δ*_H_ 4.45 (1H, dd, *J* = 6.0, 9.2 Hz, H-6) and 3.69 (1H, m, H-7), and two olefinic protons at *δ*_H_ 7.31 (1H, s, H-3) and 5.30 (1H, d, *J* = 9.2 Hz, H-5). The ^13^C NMR data (Table 1 and Figure S2) of **1** exhibits 11 carbon resonances corresponding to two methyl groups at *δ*_C_ 18.8 and 13.9, two methylene groups at *δ*_C_ 28.0 and 21.9, two oxygenated methine groups at *δ*_C_ 71.7 and 71.5, two pairs of olefinic carbons at *δ*_C_ 150.6, 139.0, 135.9 and 113.9, and one carbonyl carbon at *δ*_C_ 171.9. The ^1^H-^1^H COSY correlations of H-5/H-6/H-7/H-8 and H-1′/H-2′/H-3′ together with the key HMBC correlations from H-1′ to C-1/C-3 and from H-5 to C-3/C-4/C-6/C-7 confirmed the planar structure of **1** (Figure 2). The NOESY correlation of H-5 with H-3 indicated the *Z* configuration of the double bond in the side-chain (Figure 3). Thus, the structure of **1** was established to be an analogue of plecmillin G [7], with the main differences of the presence of an oxygenated methine group at (*δ*_H_ 3.87 (m) and *δ*_C_ 64.4 (CH)) for C-2′ in plecmillin G with a methylene group at (*δ*_H_ 1.60 (m) and *δ*_C_ 21.9 (CH_2_)) for C-2′ in **1**. 

**Figure 4:**
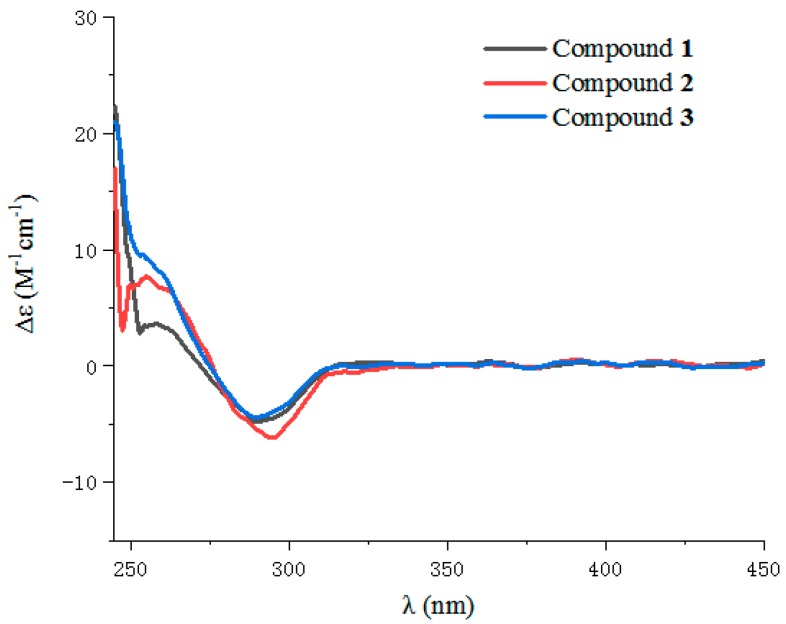
Experimental CD Spectra of **1–3**.

**Figure 5:**
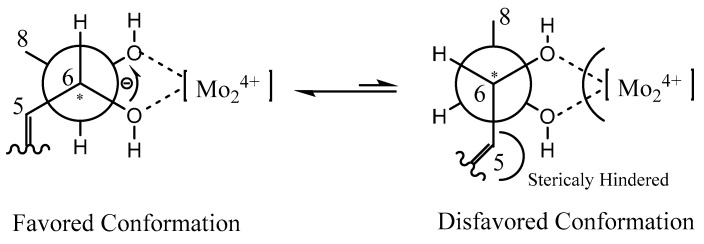
Conformations of the Mo_2_^4+^ complex of **1.**

**Figure 6:**
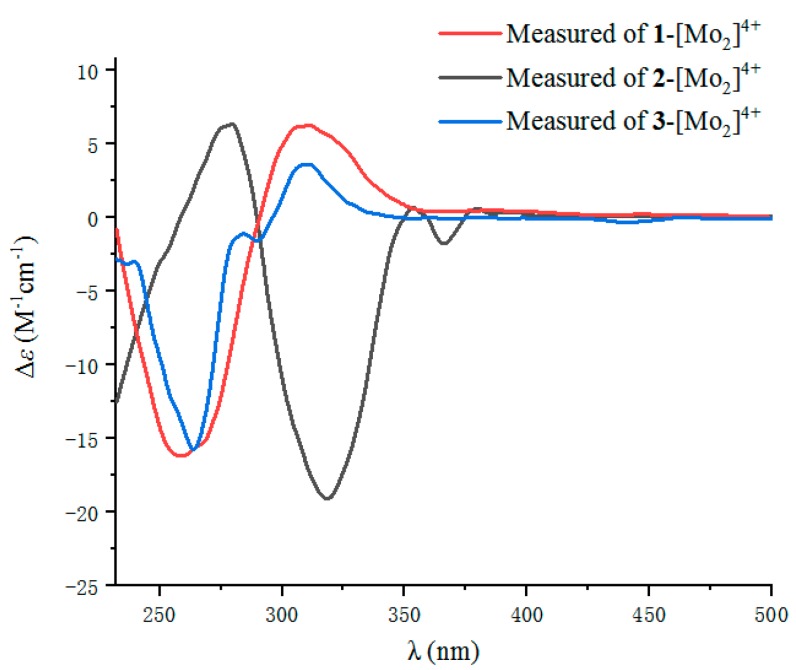
Experimental ECD spectra of the Mo_2_^4+^ complex of **1**–**3** with the inherent CD spectrum subtracted.

The absolute configuration of C-6 in **1** was determined by circular dichroism (CD) spectroscopy (Figure 4). The same negative cotton effect observed at about 280 nm for **1** suggested the *S* configuration at C-6, by comparison with data for plecmillin G [7]. Furthermore, the absolute configurations at C-6 and C-7 in **1** were determined on the basis of the CD spectrum of the complex formed in situ upon the addition of Mo_2_(OAc)_4_ to a solution of **1** in DMSO (Figure 5), the metal complex generated as an auxiliary chromophore, with the inherent CD spectra subtracted [15,16,17,18,19]. According to the empirical rule proposed by Snatzke′s method [15], the observed induced CD (ICD) curve at around 310 nm showing the same sign with the O−C−C−O torsion angle in the favored conformation allows the assignment of the absolute configuration. The ICD spectrum of the metal complex of **1** showed a positive cotton effect at 308 nm (*Δε* + 7.08) (Figure 6). Therefore, the absolute configuration of **1** was identified as 6*S*, 7*S*, and named as penicilactone A.

Compound **2** was obtained as a yellow oil, with the molecular formula of C_11_H_16_O_4_ (four degrees of unsaturation) as deduced from the HRESIMS data. Its NMR data (Table 1) were almost identical to those of **1**, the slight differences of the NMR chemical shifts (upshifted or downshifted) between **2** and **1** were clearly distinguished (Table 1). These data proposed that **2** and **1** could be sharing the same carbon skeleton, and the variations of chemical shifts for the carbons of (*δ*_C_ 113.6, 71.5, and 71.4) might be due to the presence of chiral carbons (C-6 and C-7). The planar structure was established by the HMBC and ^1^H-^1^H COSY correlations, as shown in Figure 2. The absolute configuration of C-6 in **2** was confirmed as 6*S* by the same negative cotton effect at about 280 nm as observed in (*Z*)-5-((2*S*,3*R*)-2,3-dihydroxybutylidene)furan-2(5H)-one (which was synthesized by Wang and Zhu in 2013) [7,9]. Likewise, the absolute configurations of C-6 and C-7 in **2** were also assigned using an in situ dimolybdenum CD method. The negative Cotton effect at 316 nm (*Δε* −19.3) confirmed the 6*S*, 7*R* configurations (Figure 6) [19]. Thus, compound **2** was determined and named penicilactone B.

Compound **3** was the isomer of **1**, with the molecular formula of C_11_H_16_O_4_ from the HRESIMS data. Its 1D NMR data were very similar to those of **1**, and the planar structure of **3** was assigned to be the same as that of **1** by analysis of its 1D and 2D NMR spectral data (Figure 2). The main differences between **1** and **3** were that H-3 and H-5 undergo large downfield shifts in **1** (*δ*_H-3_ 7.66 vs. 7.31, *δ*_H-5_ 5.68 vs 5.30, respectively), owing to a decrease in steric effect and C-3 experienced a large upfield shift (*δ*_C-3_ 136.4 vs. 139.0) due to steric effects between 6-CH(OH) and C-3, indicating that the double bond in the side-chain of **3** was of the *E* form [7]. The absolute configuration of C-6 in **3** was assigned as 6*S* by comparison of the specific rotation data and the cotton effects observed at about 280 nm for **3** and plecmillin I (Figure 4) [7]. The absolute configurations of C-6 and C-7 in **3** were also assigned using an in situ dimolybdenum CD method. The positive cotton effect at 308 nm (*Δε* + 4.01) (Figure 6) confirmed the 6*S*, 7*S* configurations for **3**, and the compound named penicilactone C.

### 2.2. Biological Activity

Compounds **1**–**3** were evaluated for their antibacterial activities against six pathogenic bacteria. In addition, only compound **1** showed antibacterial activity against *Staphylococcus aureus* with an MIC value of 6.25 μg/mL (Table 2). All compounds were also tested for their insecticidal activity against newly hatched larvae of *Culex quinquefasciatus*, and only **2** showed insecticidal activity against newly hatched larvae of *C. quinquefasciatus*, with an LC_50_ value of 78.5 (±0.58) μg/mL (Table 2).

## 3. Materials and Methods 

### 3.1. General Experimental Procedures

Optical rotations were measured on a JASCO P-1020 digital polarimeter (JASCO, Tokyo, Japan). Preparative HPLC were used for an Agilent 1260 prep-HPLC system with an Agilent Eclipse XDB-C18 column (9.4 × 250 mm, 7 µm, Agilent Corporation, Santa Clara, CA, USA). 1D and 2D NMR spectra were obtained on a Bruker AV-400 spectrometer (Bruker Corporation, Switzerland) (the temperature was 300 K, and the mix time of NOESY was 0.3 s) using TMS as an internal standard. The other experimental procedures were performed as reported previously [14].

### 3.2. Fungal Materials

The fungus TGM112 was isolated from the mangrove *B. sexangula var. rhynchopetala*, collected in the South China Sea in August, 2014, and was identified as *Penicillium* sp. [14]. This strain was deposited in China General Microbiological Culture Collection Center, Beijing, China, with the CGMCC code 16499. The fungal strain was cultivated in 20 L potato glucose liquid medium [14] at 25 *^◦^*C without shaking for 4 weeks.

### 3.3. Extraction and Isolation 

The fungal cultures were extracted with EtOAc (20 L, 24 h each) at three different times to obtain the extracts. The extracts were then concentrated in vacuo to yield an oily residue (20.6 g). The residue was subjected to silica gel column chromatography (CC) (petroleum ether, EtOAc *v*/*v*, gradient 100:0–0:100) to generate five fractions (Fr. 1–Fr. 5). Fr. 3 (5 g) was separated by silica gel CC and eluted with petroleum ether-EtOAc (from 10:1 to 3:1) to afford four subfractions (Fr. 3a–3d). Then Fr. 3c was separated by semi-preparative HPLC (CH_3_CN–H_2_O, 45:55 for subfraction 3c *v*/*v*) to obtain **1** (5.1 mg, *t*_R_ = 10.5 min), **2** (3.5 mg, *t*_R_ = 17.5 min), and **3** (3.2 mg, *t*_R_ = 29.1 min).

*Penicilactone* A **(1)***:* yellow oil. [*α*${]}_{D}^{24}$ −20.1 (*c* = 0.10, CHCl_3_). UV (MeOH) *λ*_max_ (log *ε*) 280 (3.02) nm; CD (*c* 2 × 10^−4^ mol/L, MeOH) *λ*max (*Δε*) 288 (−4.88) nm; IR (KBr) *ν*_max_ 3315, 3230, 1710, 1432, 1082, 798, 718 cm^−1^; ^1^H and ^13^C NMR see Table 1; HRESIMS *m*/*z* 213.1121 [M + H]^+^ (calcd. for C_11_H_17_O_4_, 213.1121).

*Penicilactone* B **(2)***:* yellow oil. [*α*${]}_{D}^{24}$ −34.3 (*c* = 0.10, CHCl_3_). UV (MeOH) *λ*_max_ (log *ε*) 280 (3.00) nm; CD (*c* 2 × 10^−4^ mol/L, MeOH) *λ*max (*Δε*) 292 (−5.65) nm; IR (KBr) *ν*_max_ 3314, 3231, 1707, 1430, 1080, 799, 720 cm^−1^; ^1^H and ^13^C NMR see Table 1; HRESIMS *m*/*z* 213.1124 [M + H]^+^ (calcd. for C_11_H_17_O_4_, 213.1121).

*Penicilactone* C **(3)***:* yellow oil. [*α*${]}_{D}^{24}$ −40.7 (*c* = 0.10, CHCl_3_). UV (MeOH) *λ*_max_ (log *ε*) 280 (3.05) nm; CD (*c* 2 × 10^−4^ mol/L, MeOH) *λ*max (*Δε*) 287(−4.98) nm; IR (KBr) *ν*_max_ 3313, 3228, 1705, 1422, 1092, 799, 716 cm^−1^; ^1^H and ^13^C NMR see Table 1; HRESIMS *m*/*z* 213.1122 [M + H]^+^ (calcd. for C_11_H_17_O_4_, 213.1121).

### 3.4. Computational Section 

According to the published procedure [18,19,20,21], compounds **1**–**3** (0.5 mg) were dissolved in a dry solution of the stock Mo_2_(OAc)_4_ complex (1.1 mg) in DMSO and then recorded immediately for the first induced ECD spectra, and its time evolution was monitored until stationary (about 30 min after mixing). 

### 3.5. Antibacterial Activities 

Antibacterial activity was determined against six pathogenic bacteria, *Escherichia coli* (ATCC 25922), *Staphylococcus aureus* (ATCC 25923), *S. albus* (ATCC 8799), *Micrococcus luteus* (ATCC 10240), *Vibrio parahaemolyticu*s (ATCC 17802) and *V. alginolyticus* (ATCC 17749) by the microplate assay method [22]. Ciprofloxacin was used as the positive control. 

### 3.6. Insecticidal Activities against Newly Hatched Larvae of Culex Quinquefasciatus

In the test, each containing 10 neonate larvaes of *C. quinquefasciatus* in 6-well microtiter plates, the examined compounds were dissolved in DMSO at the concentration of 1 mg/mL [14]. DMSO was used as the negative control, Azadirachtin was used as the positive control, and 10 mL dechlorinated water was used as the blank control. The number of dead larvae was recorded on the 1st, 2nd, 3rd, and 4th day after treatment, respectively [23].

## 4. Conclusions

In summary, three new lactones, penicilactones A−C (**1**–**3**) with a 6,7-dihydroxyocta-2,4-dien skeleton, were isolated from the mangrove-derived fungus *Penicillium* sp. TGM112. Their absolute configurations were determined by Mo_2_(OAc)_4_-induced electronic circular dichroism (ECD) and circular dichroism (CD) spectroscopy. Compound **1** showed antibacterial activity against *Staphylococcus aureus* with an MIC value of 6.25 μg/mL. Compound **2** showed insecticidal activity against newly hatched larvae of *C. quinquefasciatus* with an LC_50_ value of 78.5 (±0.58) μg/mL.

## Figures and Tables

**Table 1:** ^1^H NMR and ^13^C NMR Data (*δ*) for **1**–**3** (400/100 MHz) (*δ* in ppm, *J* in Hz) in CD_3_OD.

Position	1		2		3	
	*δ*_C_, type	*δ*_H_ (*J* in Hz)	*δ*_C_, type	*δ*_H_ (*J* in Hz)	*δ*_C_, type	*δ*_H_ (*J* in Hz)
1	171.9, C		172.0, C		172.1, C	
2	135.9, C		135.7, C		136.0, C	
3	139.0, CH	7.31, s	139.0, CH	7.32, s	136.4, CH	7.66, s
4	150.6, C		150.6, C		152.0, C	
5	113.9, CH	5.30, d (9.2)	113.6, CH	5.33, d (9.2)	113.7, CH	5.68, d (8.8)
6	71.7, CH	4.45, dd (6.0, 9.2)	71.5, CH	4.51, dd (4.4, 9.2)	72.7, CH	4.27, dd (5.2, 8.8)
7	71.5, CH	3.69, m	71.4, CH	3.80, m	71.6, CH	3.75, m
8	18.8, CH_3_	1.13, d (6.8)	18.8, CH_3_	1.15, d (6.4)	19.1, CH_3_	1.17, d (6.4)
1′	28.0, CH_2_	2.31, t (6.8)	28.0, CH_2_	2.32, t (6.8)	28.2, CH_2_	2.33, t (6.8)
2′	21.9, CH_2_	1.60, m	21.9, CH_2_	1.62, m	21.9, CH_2_	1.62, m
3′	13.9, CH_3_	0.98, t (7.2)	13.9, CH_3_	0.98, t (7.2)	13.9, CH_3_	0.98, t (7.2)

**Table 2:** Biological activities of **1**–**3**.

Compounds	MIC (µg/mL)	LC_50_ (µg/mL)
	*S. aureus*	*C. quinquefasciatus*
**1**	6.25	>80
**2**	>20.0	78.5 (±0.58)
**3**	>20.0	>80
Ciprofloxacin ^a^	0.39	
Azadirachtin ^b^		9.8 (±0.58)

^a^ Ciprofloxacin was used as a positive control. ^b^ Azadirachtin was used as a positive control.

## Supplementary Materials

The following are available online at https://www.mdpi.com/1660-3397/17/8/433/s1, Figure S1: ^1^H NMR (CD_3_OD, 400 MHz) spectrum of **1**. Figure S2: ^13^C NMR (CD_3_OD, 100MHz) spectrum of **1**. Figure S3: DEPT (CD_3_OD, 100 MHz) spectrum of **1**. Figure S4: HMQC spectrum of **1**. Figure S5: HMBC spectrum of **1**. Figure S6: ^1^H-^1^H COSY spectrum of **1**. Figure S7: NOESY spectrum of **1**. Figure S8: HRESIMS spectrum of **1**. Figure S9: ^1^H NMR (CD_3_OD, 400 MHz) spectrum of **2**. Figure S10: ^13^C NMR (CD_3_OD, 100 MHz) spectrum of **2**. Figure S11: DEPT (CD_3_OD, 100 MHz) spectrum of **2**. Figure S12: HMQC spectrum of **2**. Figure S13: HMBC spectrum of **2**. Figure S14: ^1^H-^1^H COSY spectrum of **2**. Figure S15: NOESY spectrum of **2**. Figure S16: HRESIMS spectrum of **2**. Figure S17: ^1^H NMR (CD_3_OD, 400 MHz) spectrum of **3**. Figure S18: ^13^C NMR (CD_3_OD, 100 MHz) spectrum of **3**. Figure S19: DEPT (CD_3_OD, 100 MHz) spectrum of **3**. Figure S20: HMQC spectrum of **3**. Figure S21: HMBC spectrum of **3**. Figure S22: ^1^H-^1^H COSY spectrum of **3**. Figure S23: NOESY spectrum of **3**. Figure S24: HRESIMS spectrum of **3**.
