# Supplementary material for: Bioactive Lactones from the Mangrove-Derived Fungus Penicillium sp. TGM112
Source: Mar Drugs. 2019 Jul 24;17(8):433. doi: 10.3390/md17080433 (PMC6722761; doi:10.3390/md17080433)
Supplement: Supplementary file 1 [file marinedrugs-17-00433-s001.pdf]

Supplementary Material

Bioactive Lactones from the Mangrove-Derived Fungus *Penicillium* sp. TGM112

Meng Bai [1,2], Guo-Lei Huang [1,2], Rong-Qing Mei [1,2], Bin Wang [1,2], You-Ping Luo [1,2], Xu-Hua Nong [1,2], Guang-Ying Chen [1,2,*] and Cai-Juan Zheng [1,2,*]

[1] Key Laboratory of Tropical Medicinal Resource Chemistry of Ministry of Education, College of Chemistry and Chemical Engineering, Hainan Normal University, Haikou, Hainan, 571158, People's Republic of China;

[2] Key Laboratory of Tropical Medicinal Plant Chemistry of Hainan Province, Hainan Normal University, Haikou, Hainan, 571158, People's Republic of China; xxbai2014@163.com (M.B.); huangguolei1982@hainnu.edu.cn (G.-L.H.); 15707973425@163.com (R.-Q.M.); 15180328507@163.com (W.B.); dengpengfei@gmail.com (Y.-P. L); nongxuhua4883@163.com (X.-H.N.);

* Author to whom correspondence should be addressed; E-mails: caijuan2002@163.com (C.-J.Z.) and chgying123@163.com (G.-Y.C.); Tel.: +86-898-6588-9422; Fax: +86-898-6588-9422

List of Supporting Information

Figure S1. ^{1}H NMR (CD_3OD, 400 MHz) spectrum of **1**.

Figure S2 ^{13}C NMR (CD_3OD, 100MHz) spectrum of **1**.

Figure S3 DEPT (CD_3OD, 100 MHz) spectrum of **1**.

Figure S4 HMQC spectrum of **1**.

Figure S5 HMBC spectrum of **1**.

Figure S6 ^{1}H-^{1}H COSY spectrum of **1**.

Figure S7 NOESY spectrum of **1**.

Figure S8 HRESIMS spectrum of **1**.

Figure S9 ^{1}H NMR (CD_3OD, 400 MHz) spectrum of **2**.

Figure S10 ^{13}C NMR (CD_3OD, 100 MHz) spectrum of **2**.

Figure S11 DEPT (CD_3OD, 100 MHz) spectrum of **2**.

Figure S12 HMQC spectrum of **2**.

Figure S13 HMBC spectrum of **2**.

Figure S14 ^{1}H-^{1}H COSY spectrum of **2**.

Figure S15 NOESY spectrum of **2**.

Figure S16 HRESIMS spectrum of **2**.

Figure S17 ^{1}H NMR (CD_3OD, 400 MHz) spectrum of **3**.

Figure S18 ^{13}C NMR (CD_3OD, 100 MHz) spectrum of **3**.

Figure S19 DEPT (CD_3OD, 100 MHz) spectrum of **3**.

Figure S20 HMQC spectrum of **3**.

Figure S21 HMBC spectrum of **3**.

Figure S22 ^{1}H-^{1}H COSY spectrum of **3**.

Figure S23 NOESY spectrum of **3**.

Figure S24 HRESIMS spectrum of **3**.

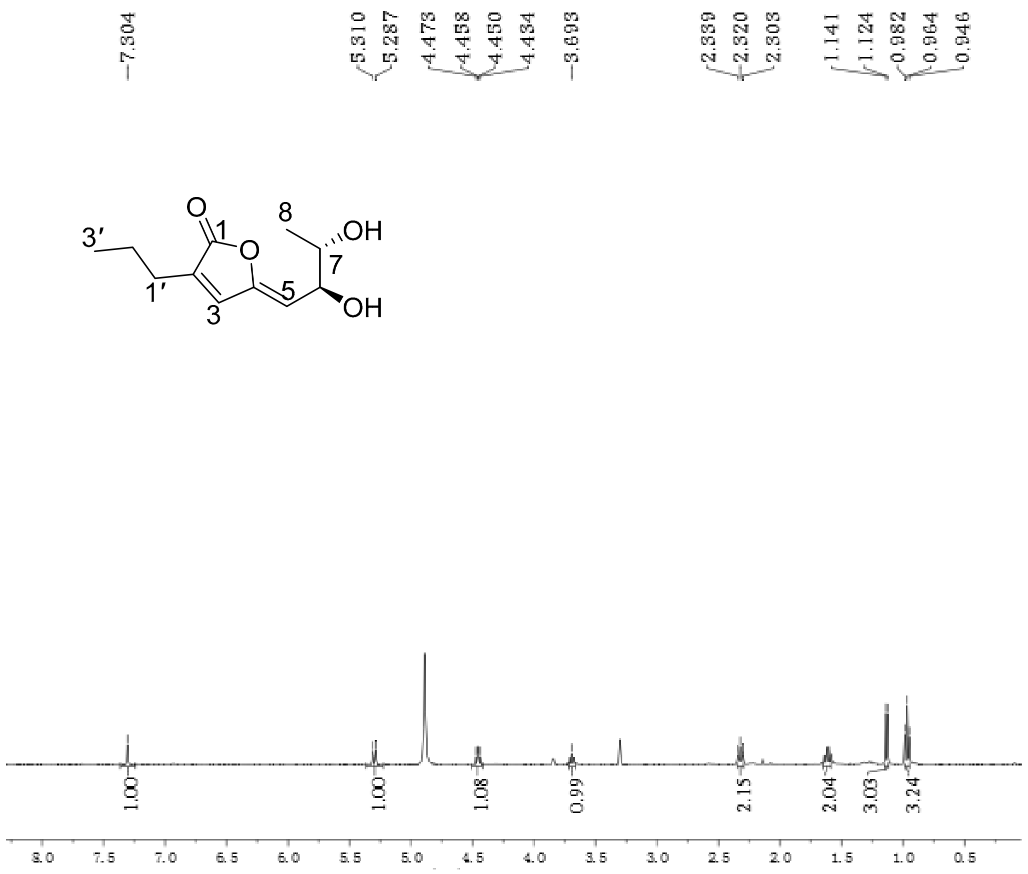

Figure S1. ^{1}H NMR (CD_3OD, 400 MHz) spectrum of **1**.

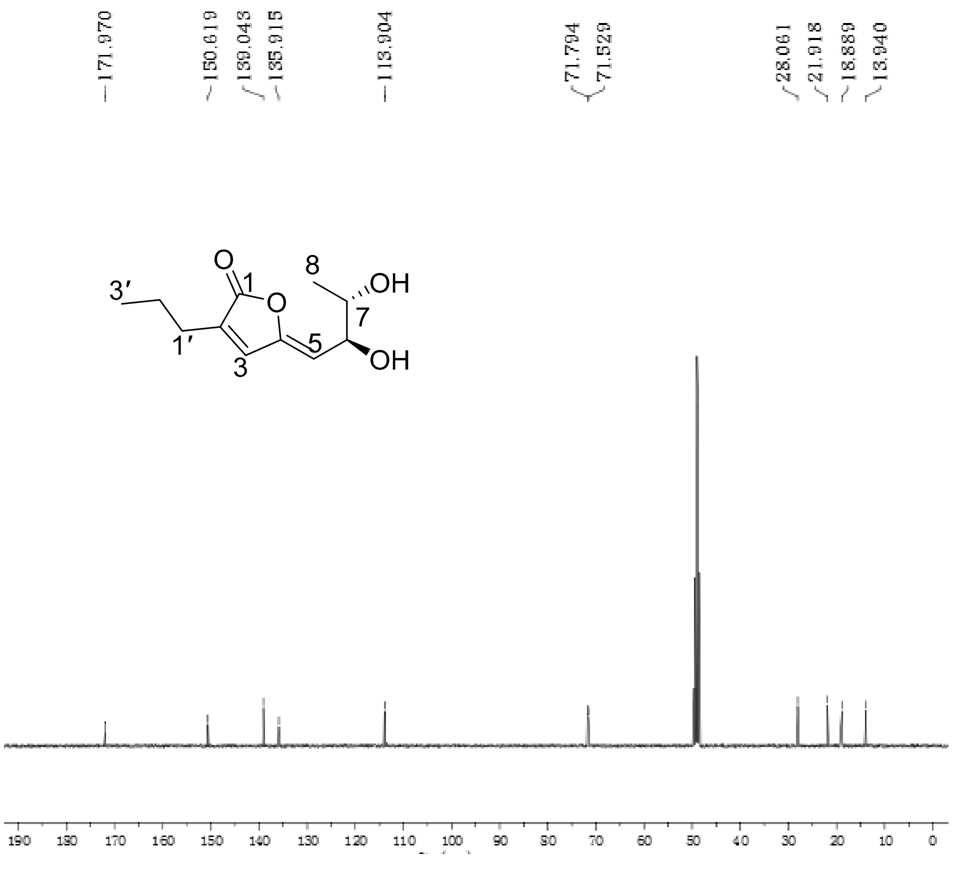

Figure S2 ^{13}C NMR (CD_3OD, 100MHz) spectrum of **1**.

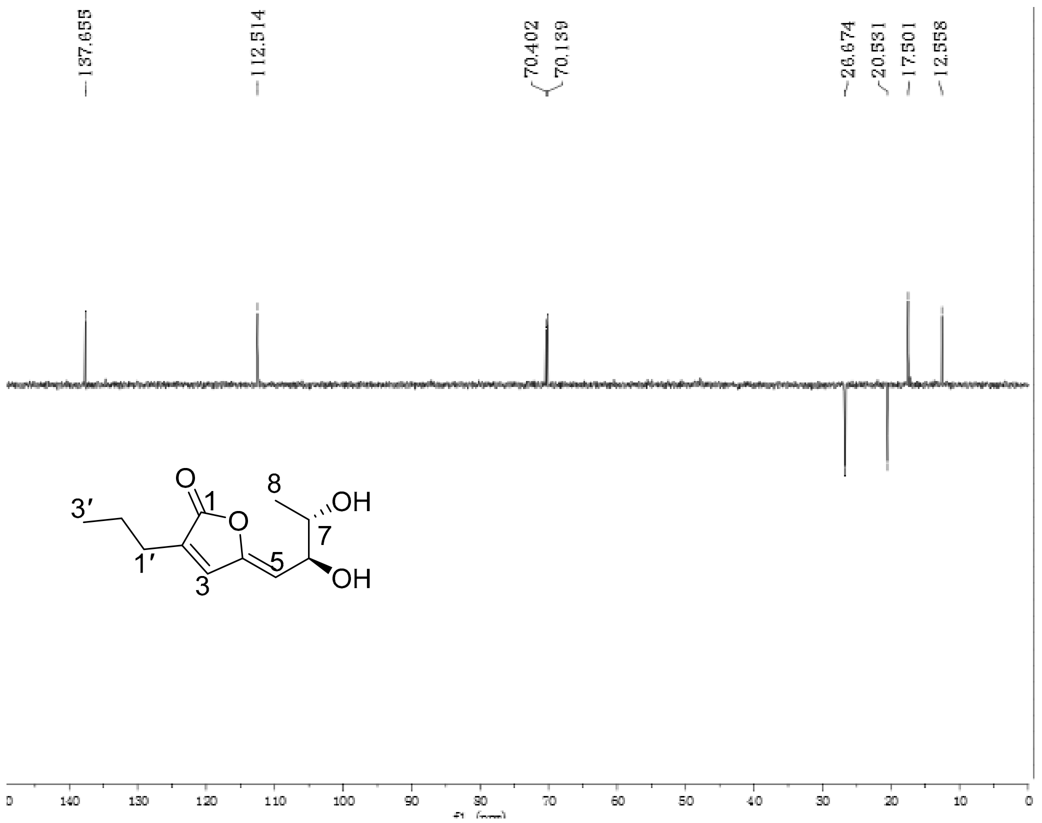

Figure S3 DEPT (CD3OD, 100 MHz) spectrum of **1**.

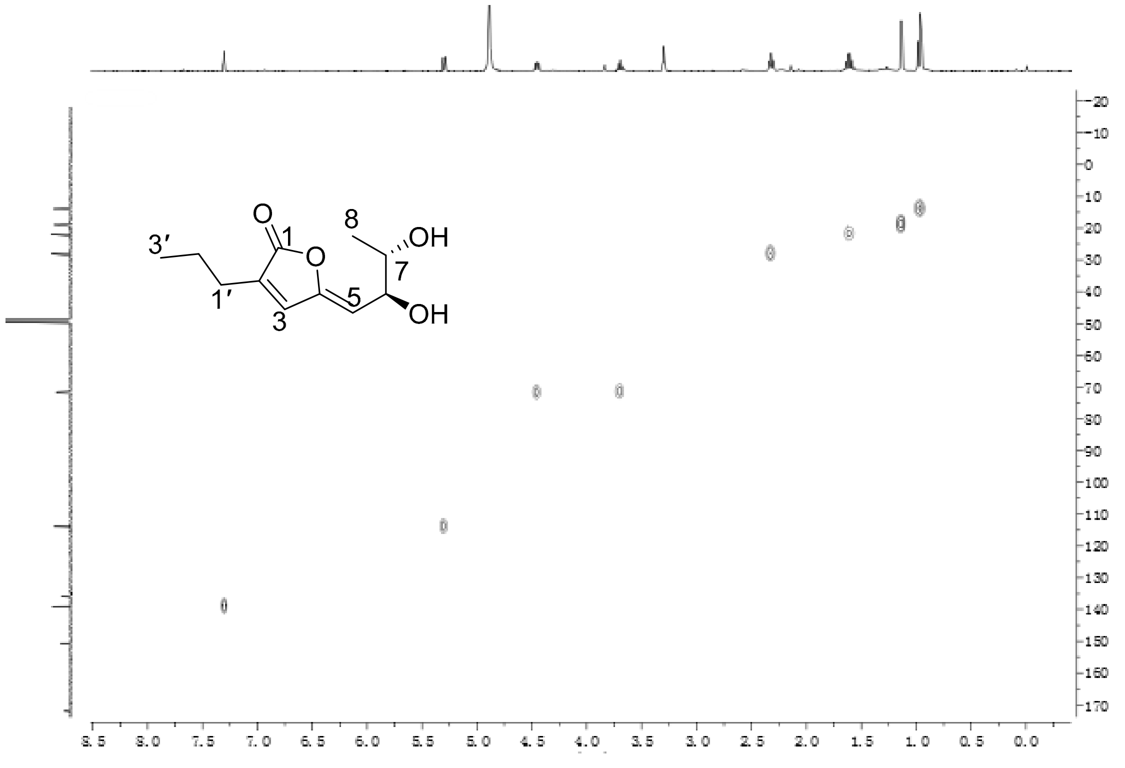

Figure S4 HMQC spectrum of **1**.

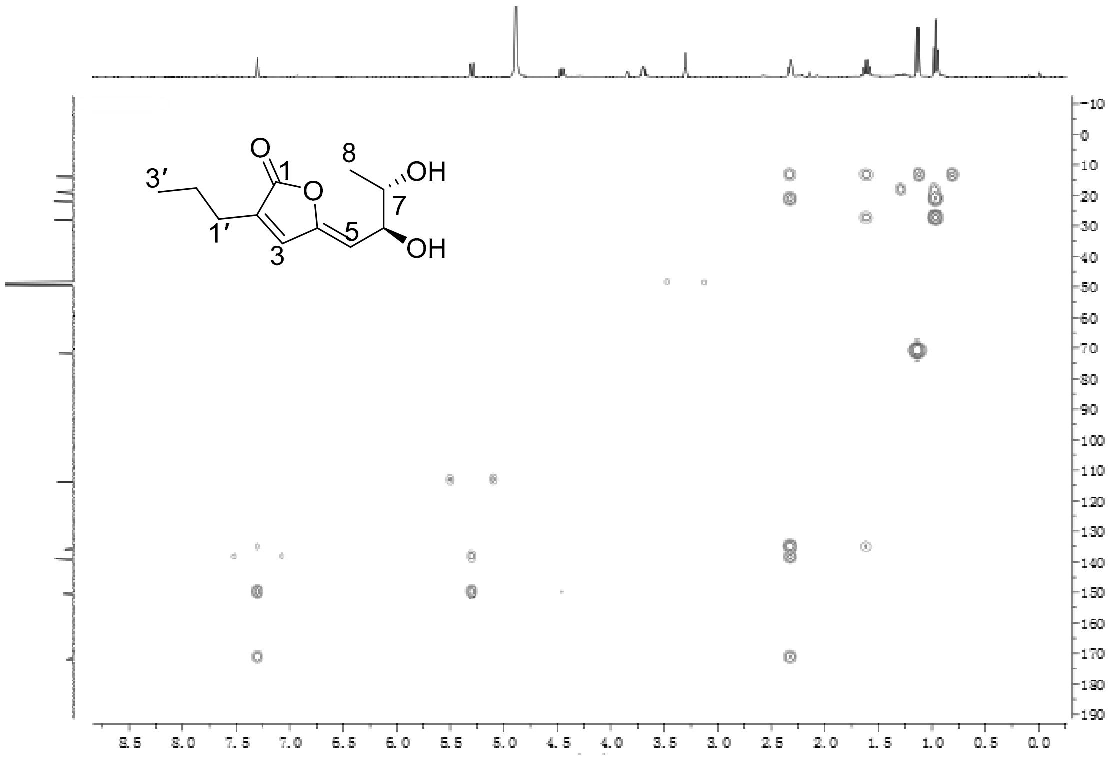

Figure S5 HMBC spectrum of **1**.

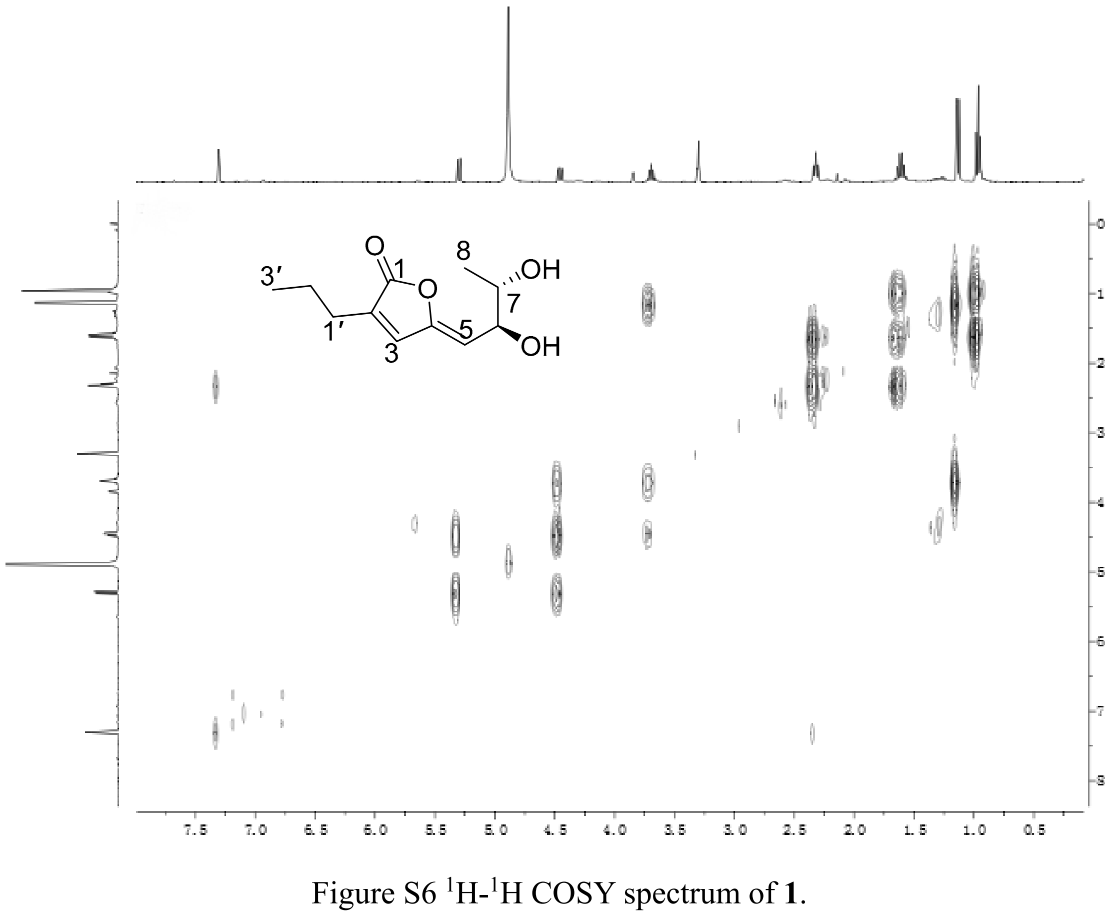

Figure S6 ^{1}H-^{1}H COSY spectrum of **1**.

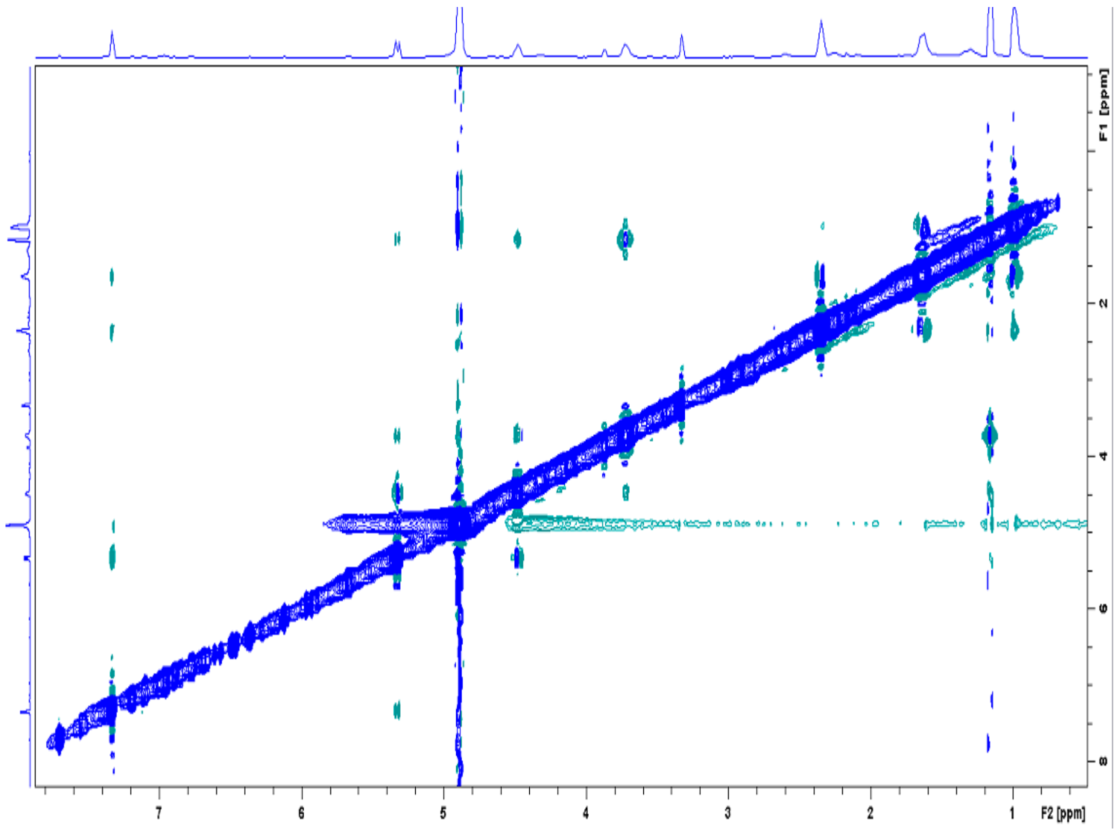

Figure S7 NOESY spectrum of **1**.

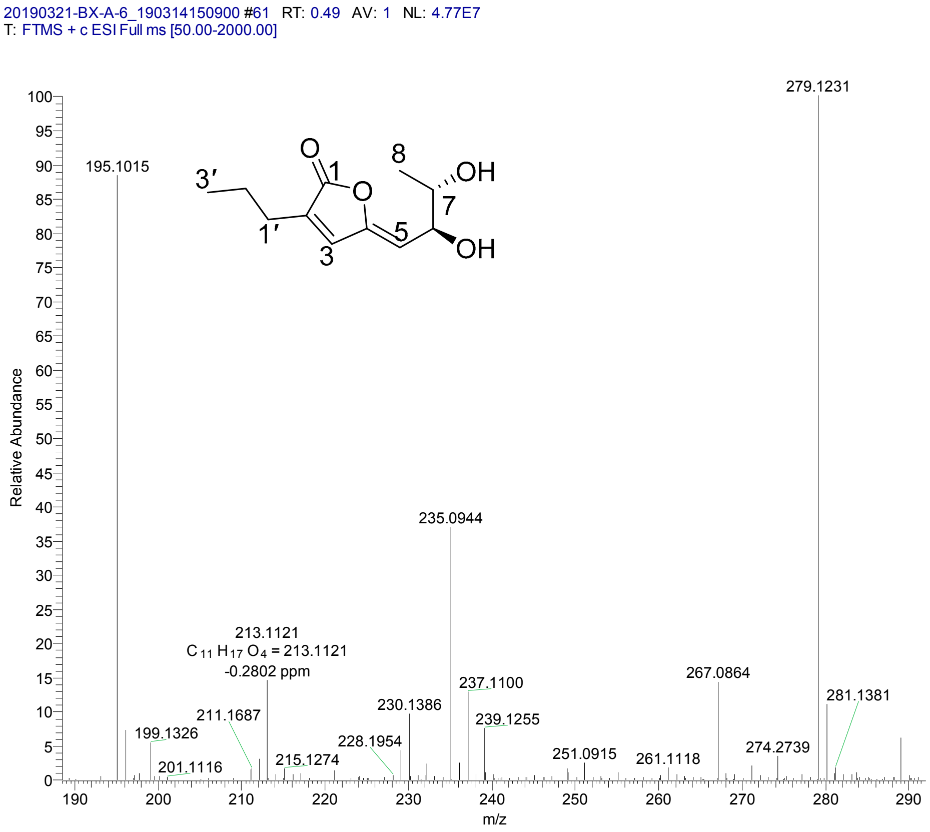

Figure S8 HR-ESI-MS spectrum of **1**.

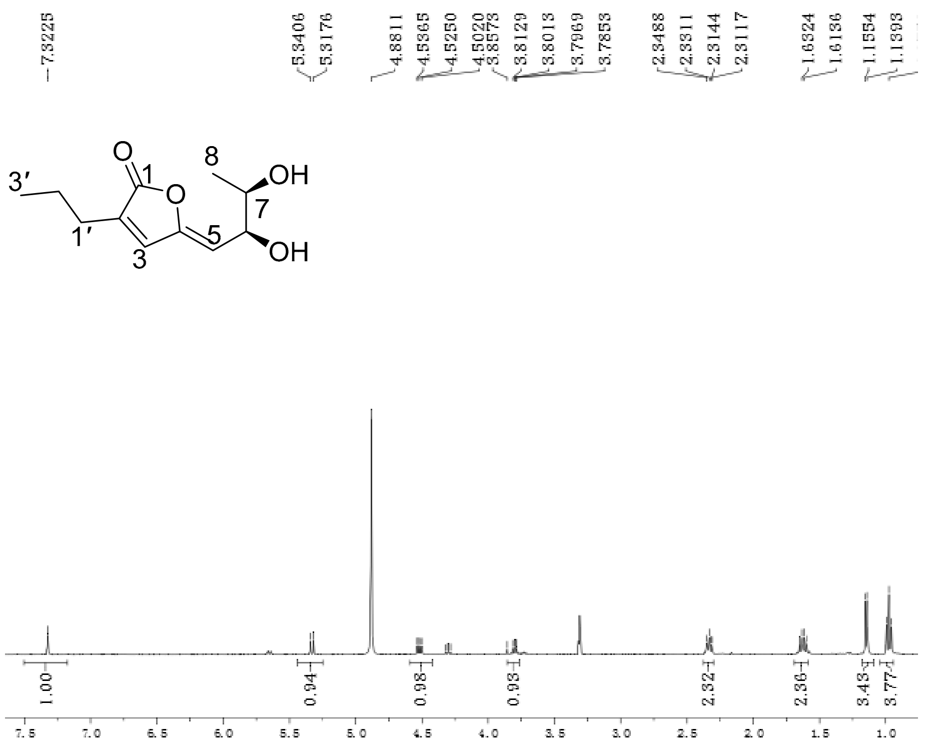

Figure S9 ^{1}H NMR (CD_3OD, 400 MHz) spectrum of **2**.

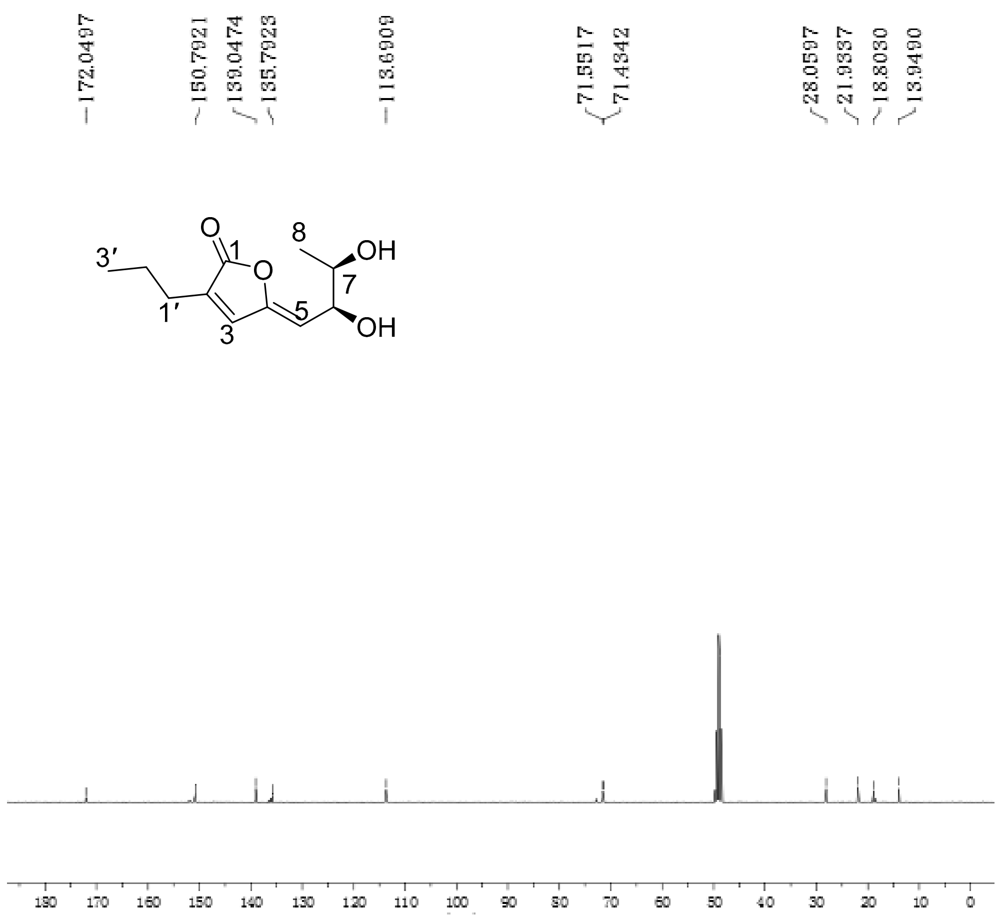

Figure S10 ^{13}C NMR (CD_3OD, 100 MHz) spectrum of **2**.

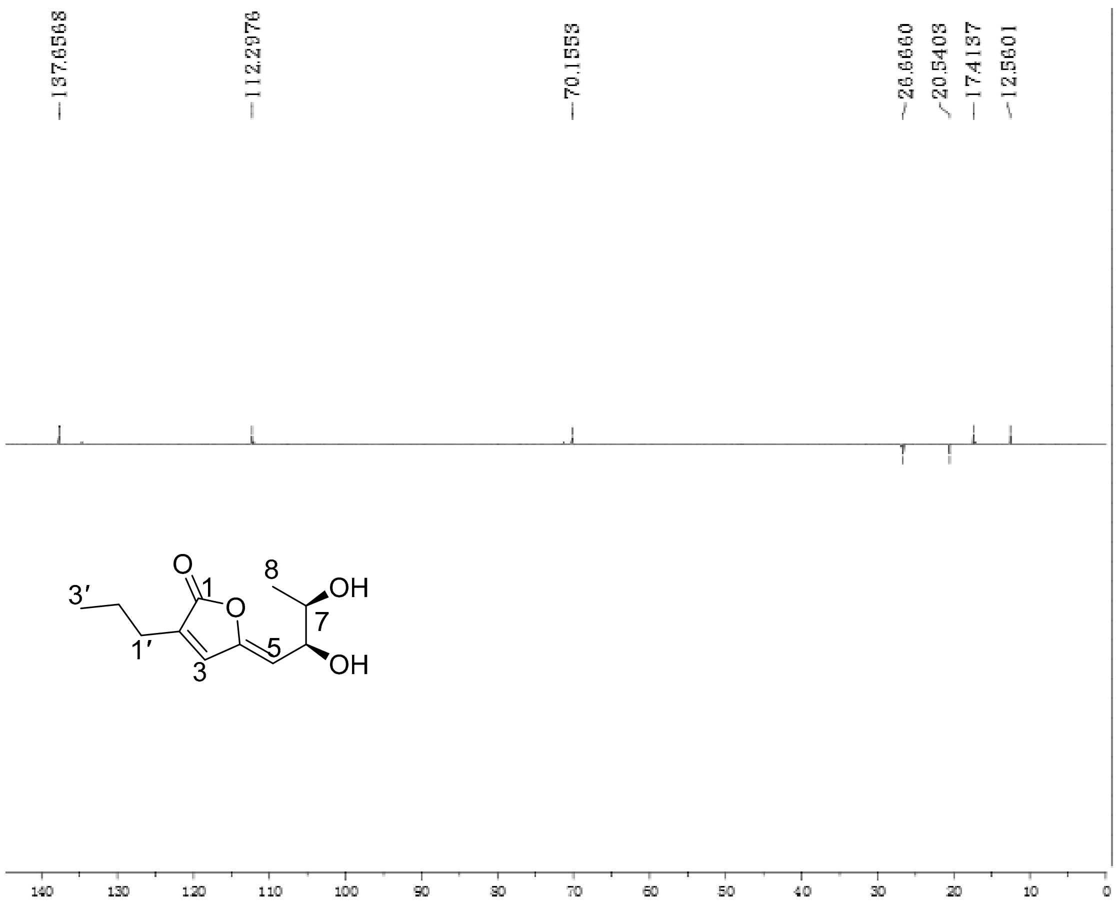

Figure S11 DEPT (CD_3OD, 100 MHz) spectrum of **2**.

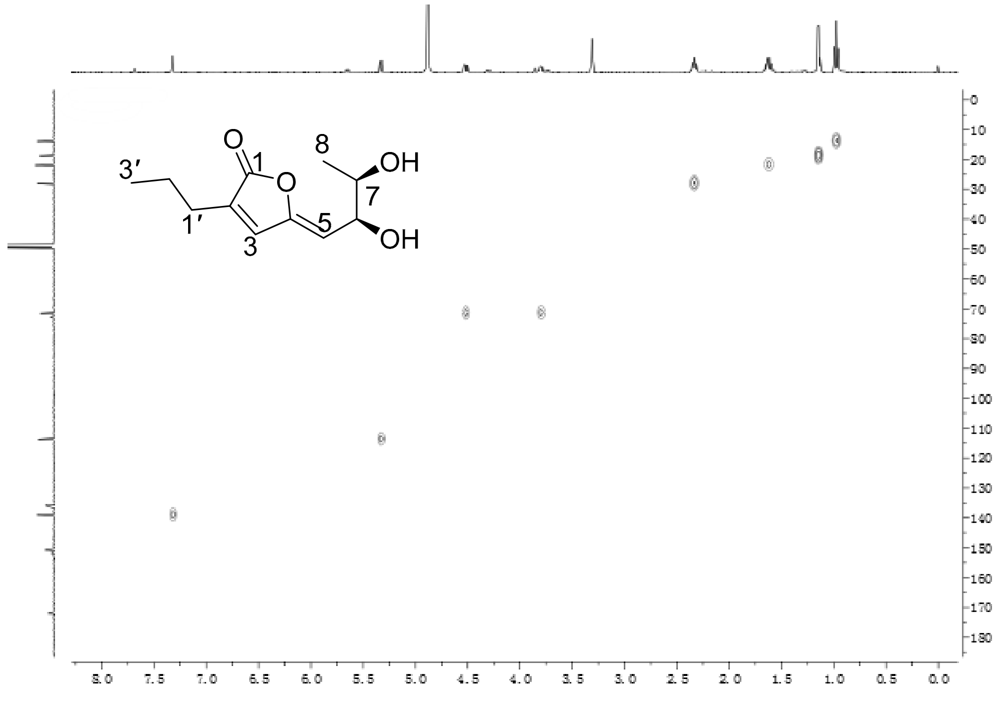

Figure S12 HMQC spectrum of **2**.

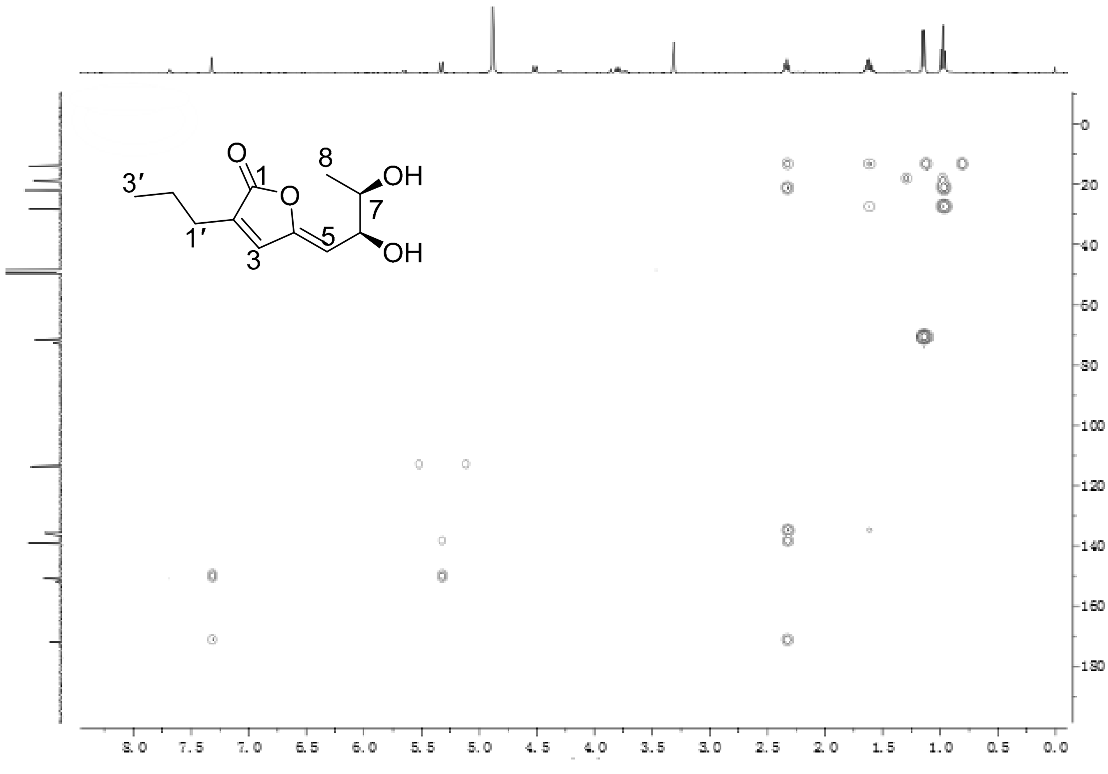

Figure S13 HMBC spectrum of **2**.

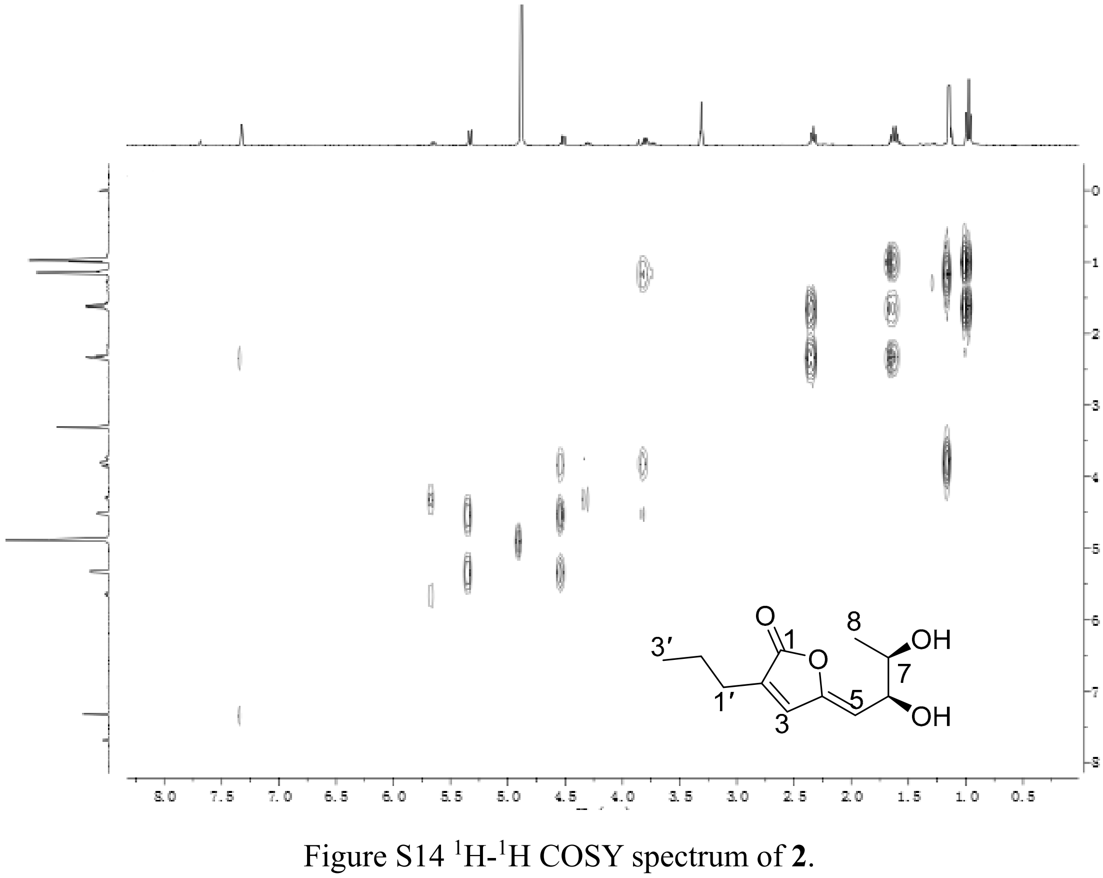

Figure S14 ^{1}H-^{1}H COSY spectrum of **2**.

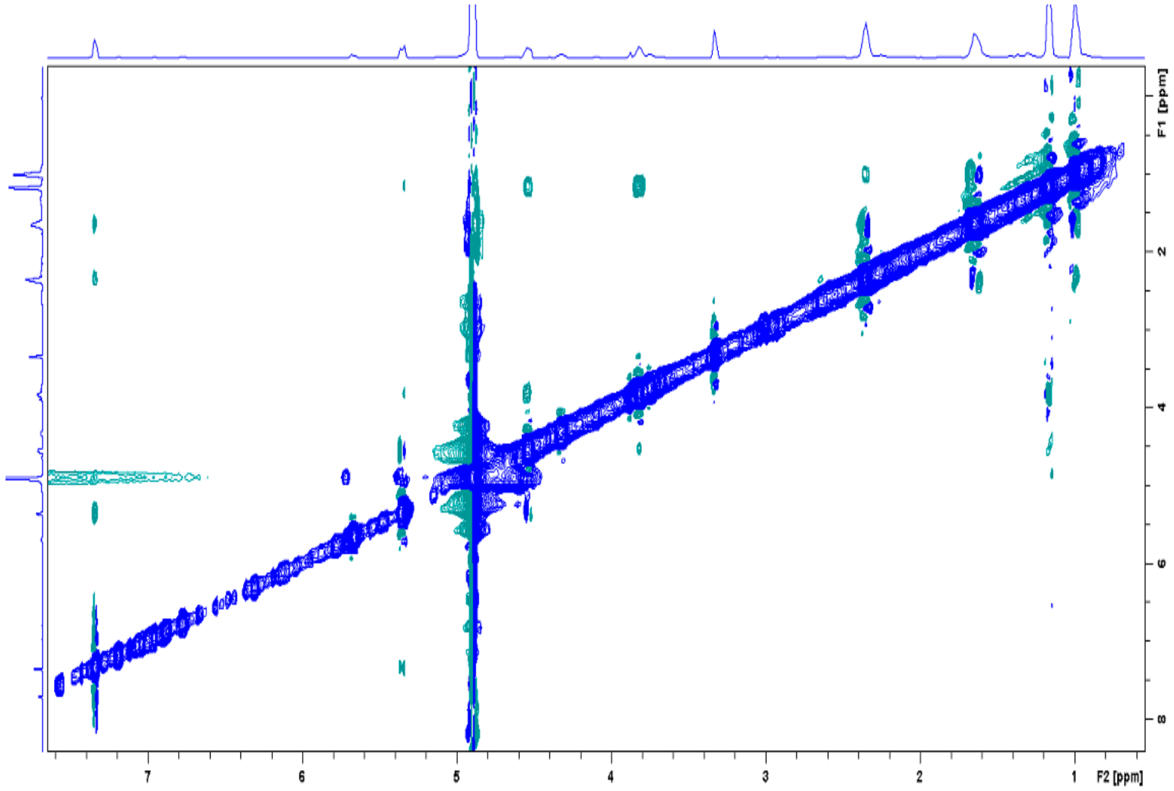

Figure S15 NOESY spectrum of **2**.

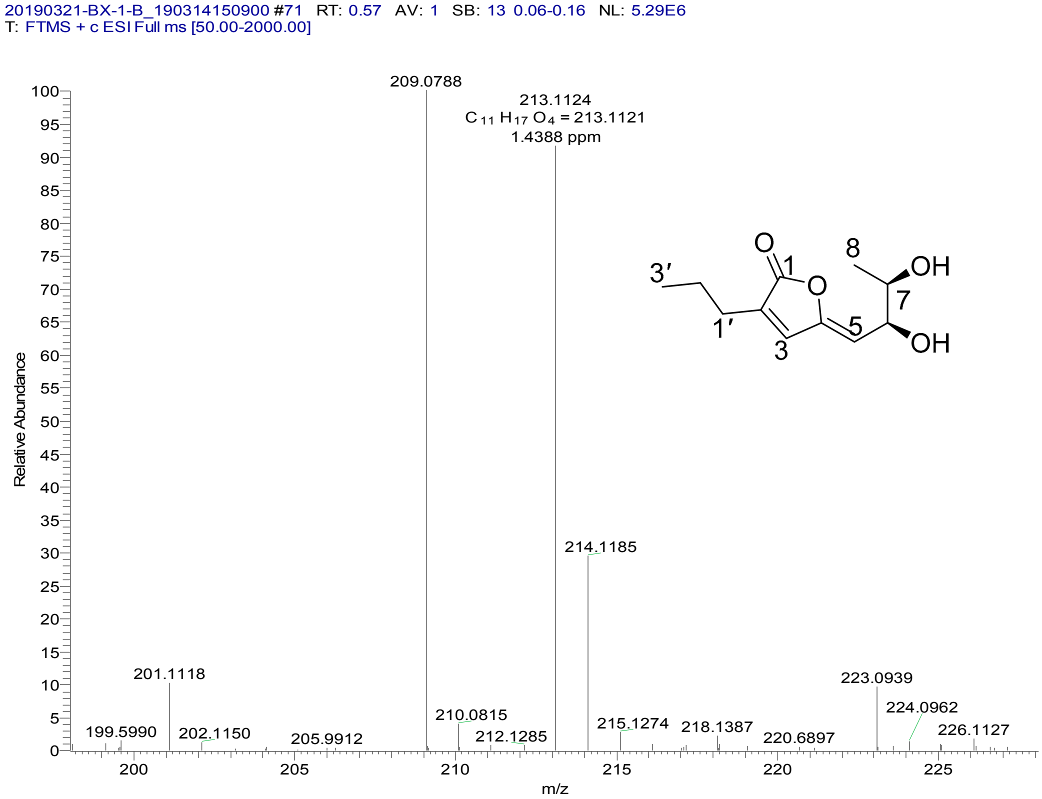

Figure S16 HR-ESI-MS spectrum of **2**.

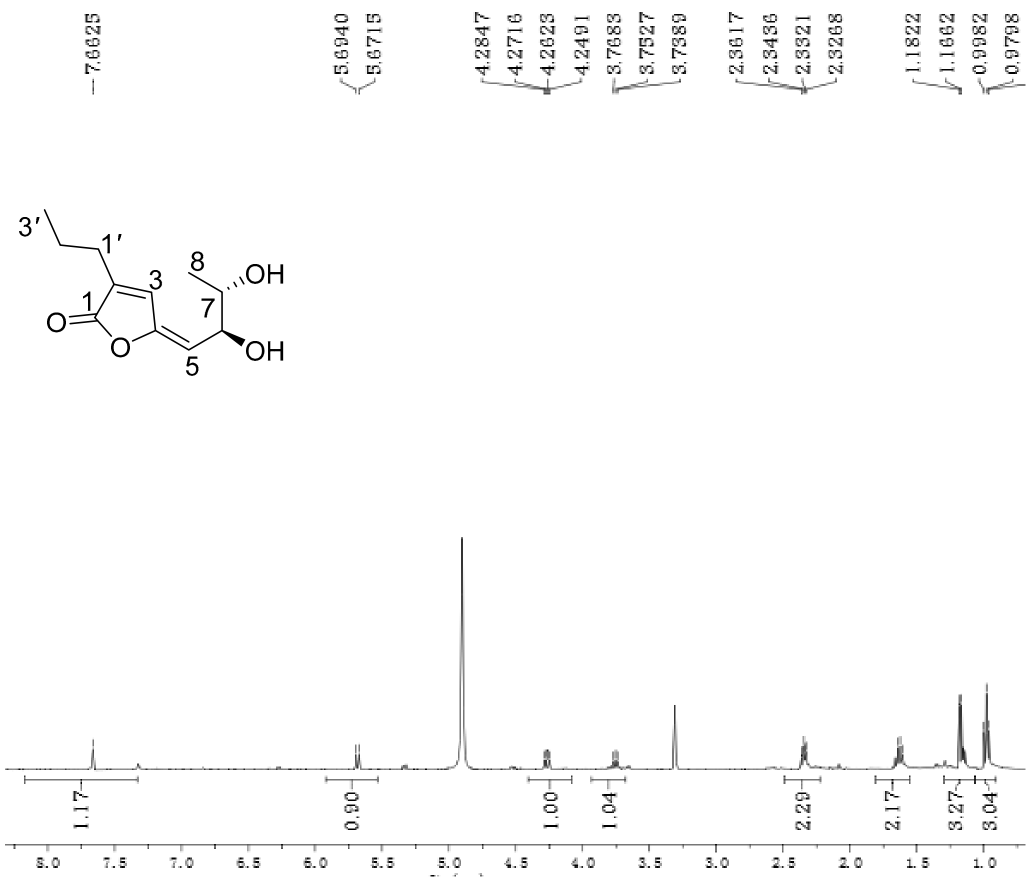

Figure S17 ^{1}H NMR (CD_3OD, 400 MHz) spectrum of **3**.

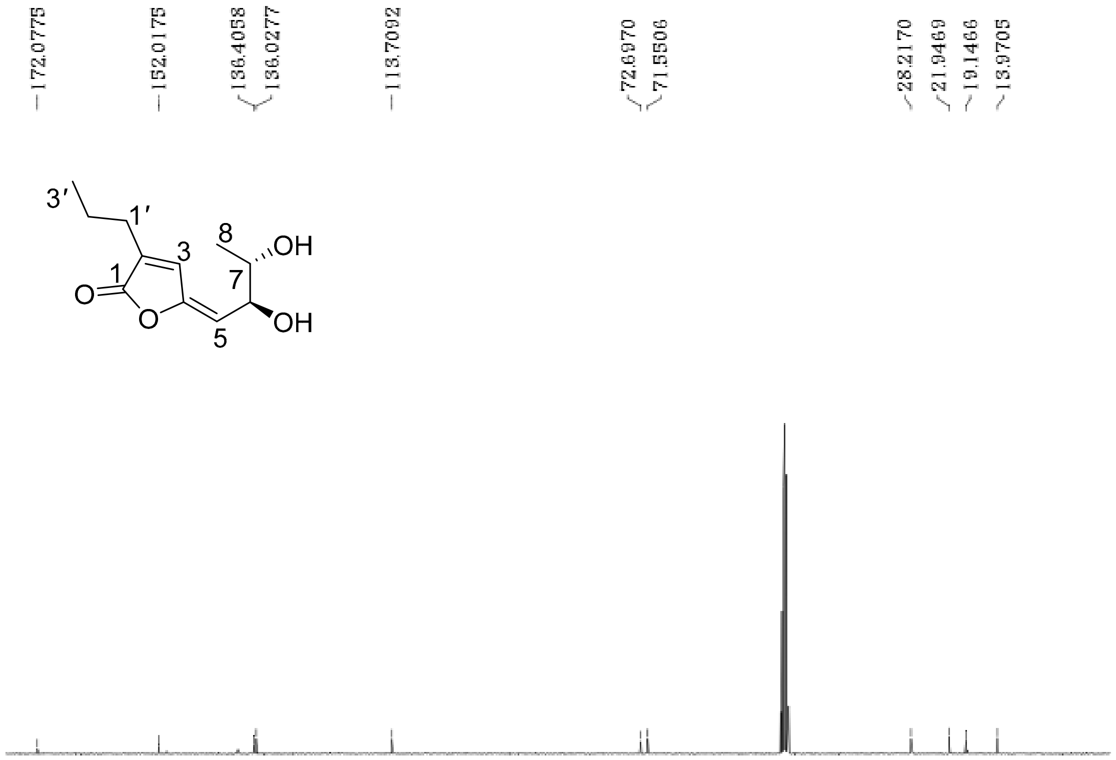

Figure S18 ^{13}C NMR (CD_3OD, 100 MHz) spectrum of **3**.

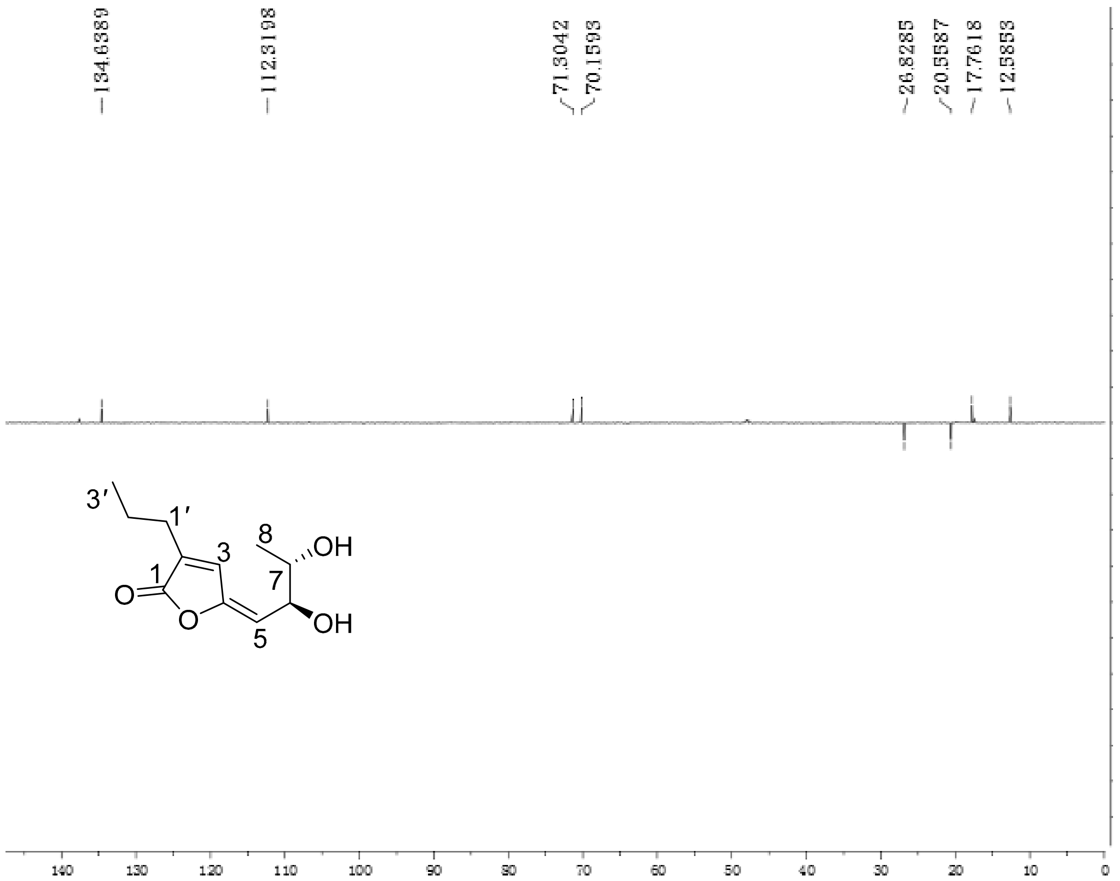

Figure S19 DEPT (CD_3OD, 100 MHz) spectrum of **3**.

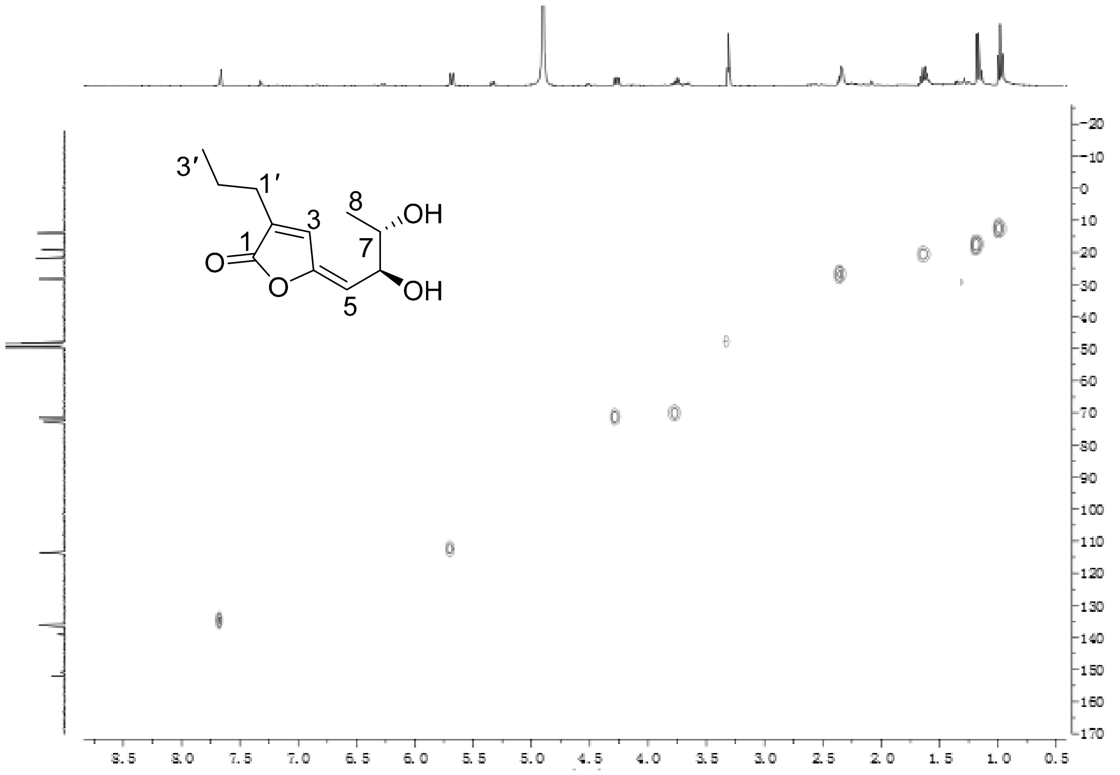

Figure S20 HMQC spectrum of **3**.

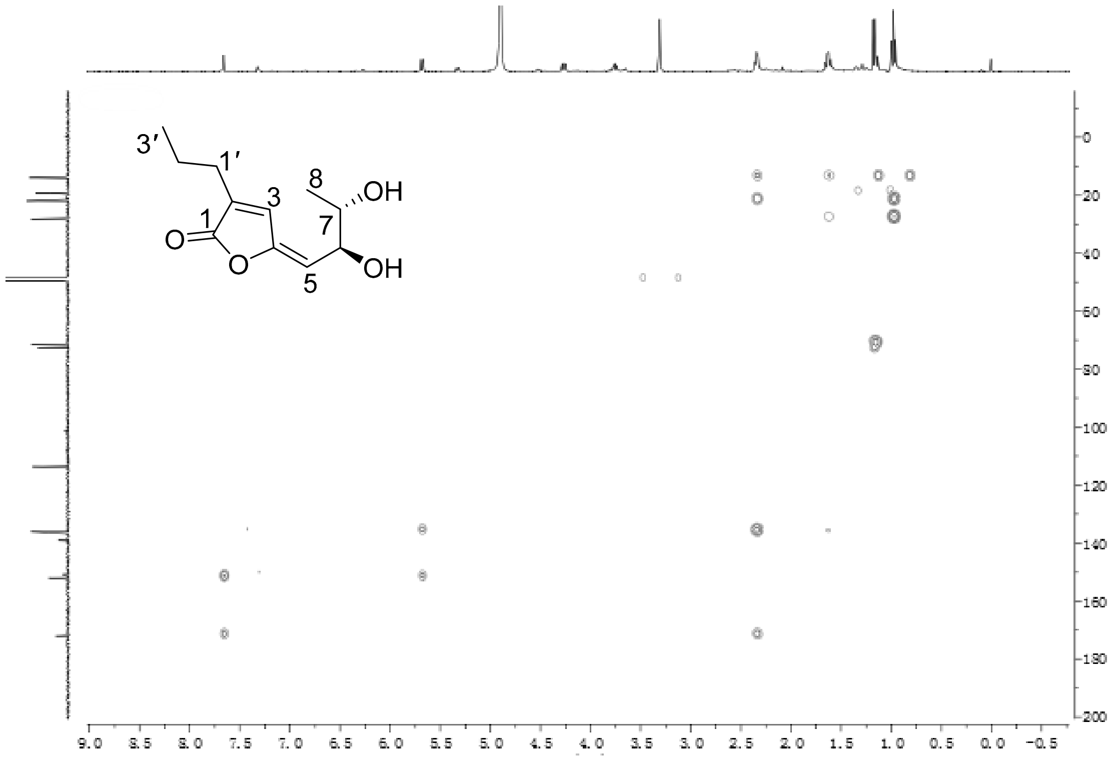

Figure S21 HMBC spectrum of **3**.

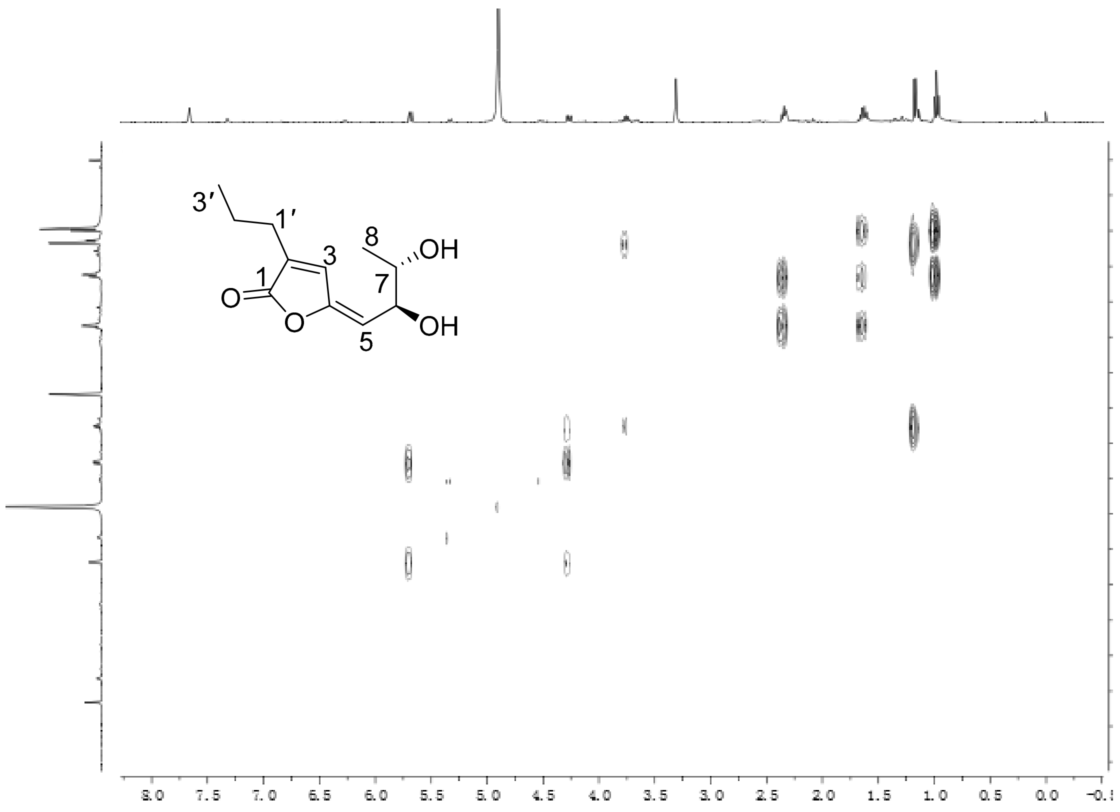

Figure S22 ^{1}H-^{1}H COSY spectrum of **3**.

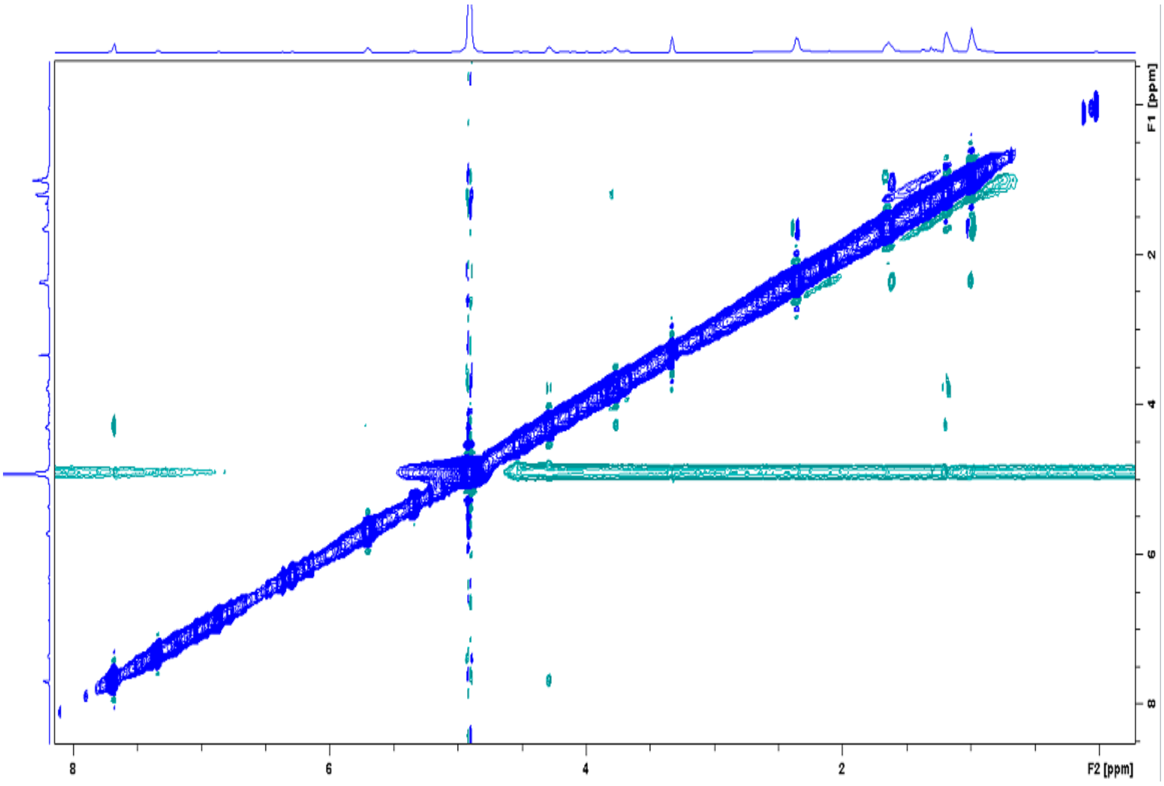

Figure S23 NOESY spectrum of **3**.

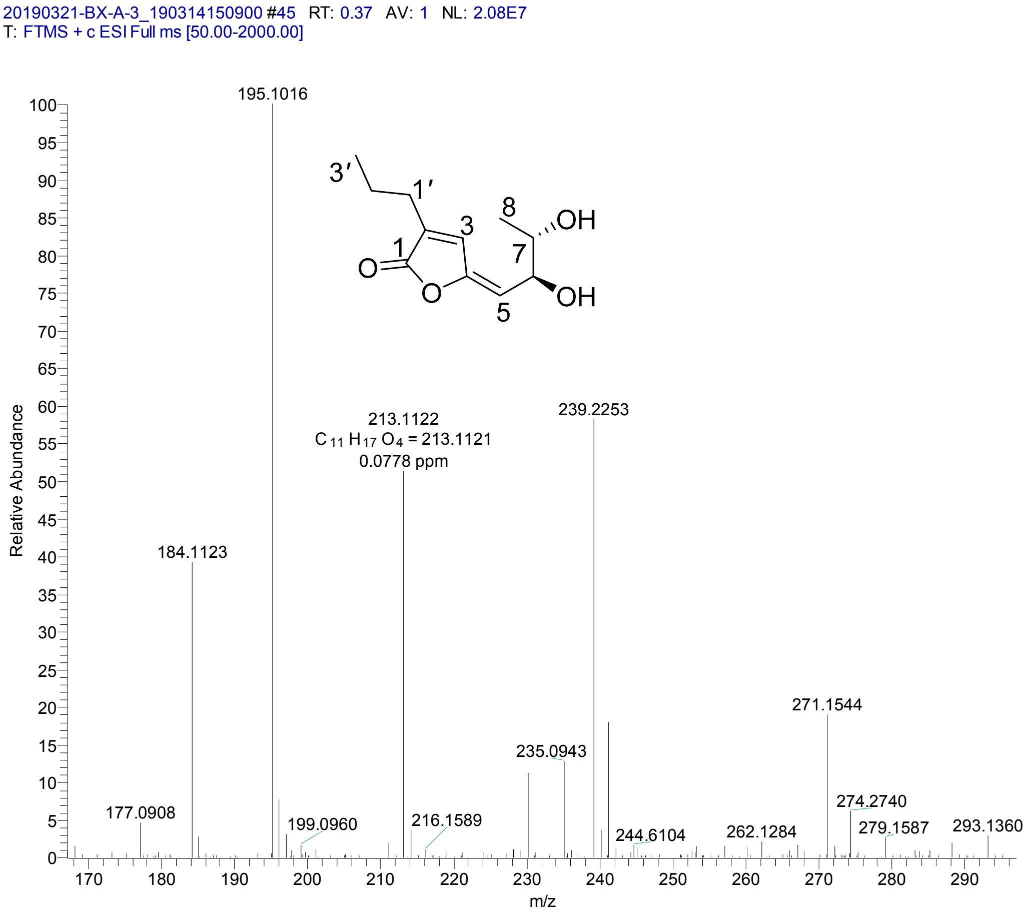

Figure S24 HR-ESI-MS spectrum of **3**.
